# Coronary Fistula–Induced Ventricular Tachycardia From Thrombotic Cyst Compression: A Case Report

**DOI:** 10.1111/anec.70121

**Published:** 2025-11-04

**Authors:** Xuelin Lu, Xiaoqin Duan, Zuoan Qin

**Affiliations:** ^1^ Department of Pathology, Changde Hospital, Xiangya School of Medicine Central South University (The First People's Hospital of Changde City) Changde China; ^2^ Department of Electrocardiogram Room, Changde Hospital, Xiangya School of Medicine Central South University (The First People's Hospital of Changde City) Changde China; ^3^ Department of Cardiology, Changde Hospital, Xiangya School of Medicine Central South University (The First People's Hospital of Changde City) Changde China

**Keywords:** coronary artery fistula, mechanical compression, myocardial fibrosis, thrombotic cyst, ventricular tachycardia

## Abstract

**Background:**

Coronary artery fistula (CAF) complicated by thrombotic cyst formation is a rare cause of ventricular tachycardia (VT), involving ischemia and mechanical compression.

**Case Presentation:**

A 55‐year‐old woman presented with palpitations, chest pain, monomorphic VT (negative V1–V6), and elevated troponin I. Imaging revealed a large right ventricular cyst. Surgical resection confirmed a thrombotic cyst communicating with a CAF. Postoperative VT recurred, leading to cardiogenic shock and death.

**Conclusions:**

CAF‐related thrombotic cysts can cause irreversible myocardial fibrosis, creating a persistent VT substrate unresponsive to anatomical intervention alone. ECG markers (fragmented QRS, ST depression) reflect irreversible damage.

## Graphical abstract

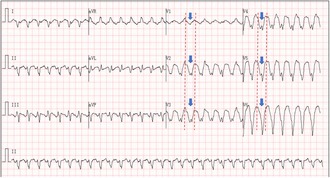

## 1 Introduction

CAFs are abnormal connections between coronary arteries and cardiac chambers or vessels. Although often asymptomatic, large CAFs can lead to complications including myocardial ischemia, heart failure, and arrhythmias through coronary steal phenomena or mass effects. We present a fatal case of CAF‐induced VT driven by dual mechanisms of chronic ischemia and mechanical compression from a thrombotic cyst, emphasizing its distinct pathophysiology and therapeutic challenges.

## 2 Case Report

**FIGURE 1:**
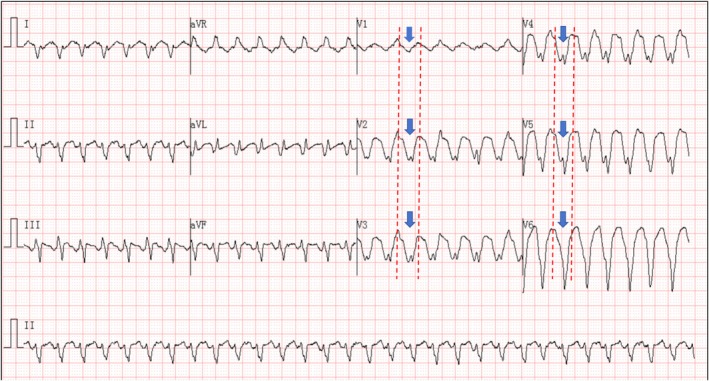
At presentation. Initial electrocardiogram: the presence of exclusively negative QRS complexes across all precordial leads (V1 through V6) supported the diagnosis of ventricular tachycardia.

**FIGURE 2:**
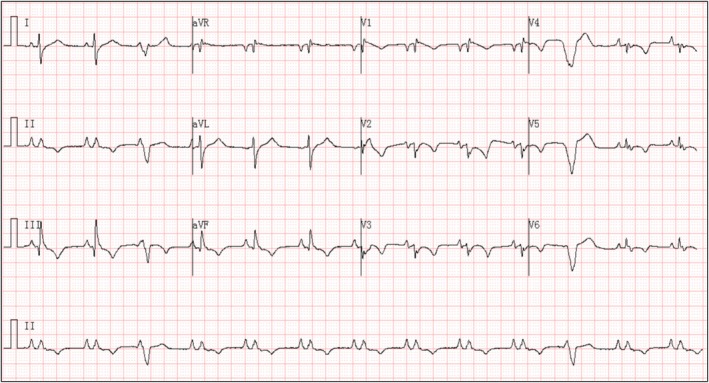
After electrical cardioversion. Electrocardiogram post‐electrical cardioversion: following successful electrical cardioversion, the ECG revealed the following findings: Peaked P waves. Ventricular premature beats. Low voltage in the precordial leads. Fragmented QRS complexes. Q wave in lead III. ST‐segment depression with T‐wave inversion in leads II, III, aVF, and V1 through V6.

A 55‐year‐old female patient without a noteworthy medical history presented to the emergency department with palpitations and chest pain. She experienced palpitations 1 month ago and developed chest pain 4 days prior to admission. Vital signs on admission were as follows: body temperature, 36.5°C; heart rate, 181 beats per minute; respiratory rate, 24 breaths per minute; blood pressure, 101/75 mmHg; and oxygen saturation, 95% in room air. An initial electrocardiogram (ECG) (Figure 1) was obtained; due to decreased blood pressure upon arrival at the emergency department, the patient underwent electrical cardioversion, after which the ECG (Figure 2) was recorded. Biochemical investigations revealed elevated troponin I, 1.000 μg/L (normal range, 0–0.023 μg/L); pro–brain natriuretic peptide, 1614 pg/mL (normal range, 0–900 pg/mL); creatine kinase‐MB isoenzyme, 32.24 U/L (normal range, 0–24 U/L); creatine kinase, 508 U/L (normal range, 40–200 U/L); and serum potassium, 4.47 mmol/L (normal range, 3.5–5.3 mmol/L). Transthoracic echocardiography revealed a cystic mass measuring approximately 94 × 69 mm in the right ventricle, a left‐to‐right shunt across the atrial septum with a width of approximately 5 mm, and pericardial effusion with a maximum posterior pericardial depth of 11 mm. Chest computed tomography (CT) suggested a large cardiac mass.

The preliminary diagnoses were (1) Cardiac mass; (2) VT. On the second day of admission, the patient underwent cardiac tumor resection, atrial septal defect repair, and tricuspid valvuloplasty. Intraoperatively, a cyst (9 × 6 × 8 cm) containing a thrombus was found in the myocardium of the right ventricular inferior wall, along with a coronary artery fistula (with a 0.5 cm orifice communicating with the cyst cavity) and an ostium secundum atrial septal defect (1 × 1 cm). A postoperative pathological examination confirmed the presence of a thrombotic cyst with myocardial fibrosis. Postoperative cardiac function was monitored serially by echocardiography. The examination on postoperative Day 9 revealed a preserved left ventricular ejection fraction (LVEF of 72%). One month after surgery, echocardiography indicated clinical progression, evidenced by significant right atrial enlargement, and a decline in LVEF to 56%. The discharge medication regimen included metoprolol succinate (23.75 mg/day), spironolactone (20 mg/day), and furosemide (20 mg/day) for ongoing management of heart failure and arrhythmia risk. Amiodarone was withheld based on the rationale of sustained postoperative rhythm stability and the well‐established risk of long‐term pulmonary toxicity associated with its use. We have added discharge medication information in the case report section. Three months after the surgery, the patient was readmitted because of recurrent palpitations and dyspnea. Recurrent episodes of VT progressed to cardiogenic shock and multiple organ failure, eventually leading to death.

This case indicates that such lesions carry a persistent risk of inducing arrhythmia. The following is an analysis of the key electrocardiographic mechanisms. Initial ECG (Figure 1): All QRS complexes in the chest leads V1through V6 were negative, which is consistent with the characteristics of VT (Vereckei et al. 2023). The mechanism may be related to the formation of abnormal conduction pathways due to myocardial fibrosis around the fistula (Bhalgat et al. 2018); the coronary artery fistula causes chronic ischemia in the local myocardium, thereby activating reentrant circuits. Post‐cardioversion ECG (Figure 2): Fragmented QRS complexes appeared in multiple leads, reflecting local conduction delay caused by cyst compression and myocardial fibrosis (Canpolat et al. 2013); peaked P waves suggested increased right atrial load (possibly secondary to compression by the right ventricular mass); extensive ST‐segment depression with T‐wave inversion (in leads II, III, aVF, V1 through V6) was attributed to subendocardial ischemia resulting from the large intracardiac cyst impeding ventricular diastolic function.

**FIGURE 3:**
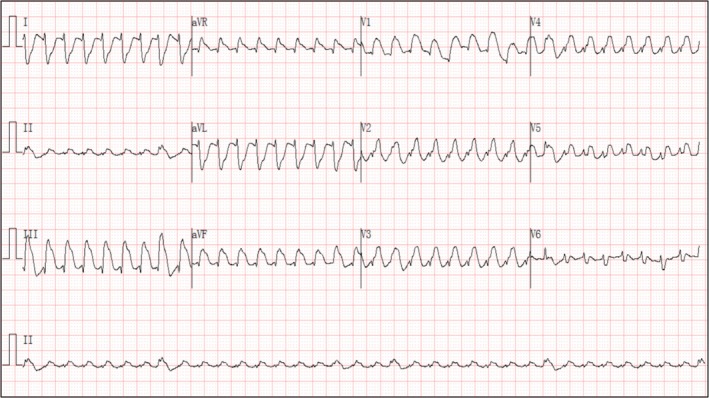
ECG during readmission. ECG during readmission: The recurrent VT remained monomorphic, the recurrent VT demonstrated a left bundle branch block (LBBB) pattern with a superior axis. We present a fatal case of CAF‐induced VT driven by dual mechanisms of chronic ischemia and mechanical compression from a thrombotic cyst, emphasizing its distinct pathophysiology and therapeutic challenges.

ECG during readmission (Figure 3): The recurrent ventricular tachycardia exhibited a left bundle branch block morphology with a superior axis, indicating an origin site or exit in the right ventricular anteroseptal or apical region, distinct from the initial VT; this change in morphology underscores the presence of an extensive and heterogeneous arrhythmogenic substrate within the right ventricle. The mechanism is attributed to persistent myocardial fibrosis and chronic ischemia resulting from the coronary steal phenomenon of the residual fistula, which created a proarrhythmic substrate capable of sustaining different reentrant circuits even after surgical removal of the cyst.

## 3 Interpretation

Initial ECG (Figure 1) showed downward QRS complexes in the precordial leads (V1 through V6), supporting the diagnosis of ventricular tachycardia (VT). The post‐cardioversion ECG (Figure 2) demonstrated peaked P waves, ventricular premature beats (with morphology consistent with those during tachycardia), low‐voltage QRS complexes in the chest leads, fragmented QRS complexes, a q wave in lead III, and ST‐segment depression with T‐wave inversion in multiple leads (II, III, AVF, V1 through V6). The recurrent VT remained monomorphic; however, its morphology differed from the initial episode. The recurrent VT (Figure 3) demonstrated a left bundle branch block (LBBB) pattern with a superior axis.

## 4 Discussion

A coronary artery fistula with a secondary thrombotic cyst leading to fatal VT is characterized by dual pathological mechanisms: (1) Anatomical compression effect: A large cyst directly impairs ventricular relaxation, inducing extensive post‐cardioversion ST segment depression and T‐wave inversion (subendocardial ischemia). Concurrently, compression leads to increased right atrial load, resulting in peak P waves. Late VT storms associated with intramyocardial dissecting hematomas of the left ventricle have been reported (Iqbal et al. 2024). (2) Arrhythmogenesis due to ischemic fibrosis (Disertori et al. 2016): The coronary steal phenomenon caused by a fistula leads to chronic local myocardial ischemia, and fibrosis around the fistula forms abnormal conduction pathways. This is manifested by characteristic changes in the initial VT ECG (uniformly negative QRS complexes in chest leads V1 through V6), whereas the presence of fragmented QRS complexes in multiple leads after cardioversion further confirms the conduction delay caused by myocardial fibrosis.

Despite surgical resection of the mass and repair of the atrial septal defect, the patient died 3 months postoperatively due to recurrent VT that progressed to cardiogenic shock and multiple organ failure. This suggests that, although the mass was removed, the arrhythmogenic substrate of the fibrotic myocardium remained. Ischemia from residual microfistulas or surgical trauma may activate new reentrant circuits, and the possibility of recurrent cyst thrombosis causing right ventricular compression leading to VT storms cannot be excluded.

The clinical course of this case differs significantly from that of fulminant myocarditis. Although both coronary artery fistulas with secondary thrombotic cysts and fulminant myocarditis can cause VT and cardiogenic shock, there are fundamental differences between them (1) Pathological basis and ECG evolution: Fulminant myocarditis is characterized by diffuse myocardial inflammation, with ECG typically showing transient ST‐segment elevation (Yang et al. 2022), which is reversible as the inflammation resolves. In contrast, the present case involved a localized fibrotic mass, and the ECG showed persistent repolarization abnormalities (such as extensively fragmented QRS complexes after cardioversion), reflecting irreversible conduction disturbances. (2) Biomarkers and imaging: In patients with fulminant myocarditis, troponin I is often markedly elevated (> 50 μg/L), and echocardiography shows diffuse wall motion hypokinesis; in this case, however, troponin was only moderately elevated (1.000 μg/L), and imaging clearly indicated a right ventricular mass with local compression effects. (3) Treatment response and prognosis: Survivors of fulminant myocarditis often regain sinus rhythm after supportive therapy (Ginsberg and Parrillo 2013), in which case, even after surgical resection of the cyst, VT recurs because of the residual arrhythmogenic substrate, eventually progressing to multiple organ failure. This highlights the limitations of anatomical intervention in correcting electrophysiological abnormalities. The core difference between the two lies in the fact that malignant arrhythmias in fulminant myocarditis stem from reversible inflammatory reactions, whereas coronary artery fistula‐related masses continuously induce electrical abnormalities through permanent structural damage (fibrosis and mechanical compression).

We acknowledge that the precise characterization of the arrhythmogenic substrate (e.g., scar‐related reentry) remains inferential in the absence of postoperative cardiac magnetic resonance or electrophysiological study. Nevertheless, by synthesizing the distinct morphology of the recurrent VT, the absence of significant left coronary stenosis on preoperative angiography, and the longitudinal deterioration in cardiac function, we strengthen the argument that an irreversible electrophysiological substrate established by pre‐existing fibrosis and chronic ischemia persisted despite anatomical correction. The resulting cardiogenic shock was likely multifactorial, primarily driven by recurrent, refractory VT, with contributions from progressive biventricular dysfunction.

## 5 Conclusions

CAF‐related thrombotic cysts can cause irreversible myocardial fibrosis, creating a persistent VT substrate unresponsive to anatomical intervention alone. ECG markers (persistent fragmented QRS, diffuse ST depression) reflect irreversible structural damage, distinguishing this entity from reversible conditions such as acute fulminant myocarditis.

## Author Contributions

**Zuoan Qin:** study conceptualization; writing – original draft; final approval. **Xuelin Lu:** investigation, writing – original draft; data collection. **Xiaoqin Duan:** methodological analysis conceptualization. All authors cared about patients and approved the final manuscript.

## Ethics Statement

The study was approved by the ethical committee of The First People's Hospital of Changde City followed by the ethical declaration of Helsinki.

## Conflicts of Interest

The authors declare no conflicts of interest.

## Supporting information


**Figure S1:** Cardiac CT Showing Large Right Ventricular Mass.
**Figure S2:** Right Coronary Angiography Showing Aneurysm and Occlusion.

## Data Availability

The data that supports the findings of this study are available in the Supporting Information of this article.
